# Variants in Neurotransmitter-Related Genes Are Associated with Alzheimer’s Disease Risk and Cognitive Functioning but Not Short-Term Treatment Response

**DOI:** 10.3390/neurolint17050065

**Published:** 2025-04-24

**Authors:** Tirso Zúñiga-Santamaría, Blanca Estela Pérez-Aldana, Ingrid Fricke-Galindo, Margarita González-González, Zoila Gloria Trujillo-de los Santos, Marie Catherine Boll-Woehrlen, Rosalía Rodríguez-García, Marisol López-López, Petra Yescas-Gómez

**Affiliations:** 1 Department of Genetics, Instituto Nacional de Neurología y Neurocirugía Manuel Velasco Suárez, Mexico City 14269, Mexico; tirzozzu@hotmail.com; 2 Master’s Program in Pharmaceutical Sciences, Universidad Autónoma Metropolitana Unidad Xochimilco, Mexico City 04960, Mexico; blankita0807@gmail.com; 3 HLA Laboratory, Instituto Nacional de Enfermedades Respiratorias Ismael Cosío Villegas, Mexico City 04530, Mexico; ingrid_fg@yahoo.com.mx; 4 Unidad de Cognición y Conducta, Instituto Nacional de Neurología y Neurocirugía Manuel Velasco Suárez, Mexico City 14269, Mexico; magogonzalez85@hotmail.com; 5 Departamento de Geriatría, Instituto Nacional de Neurología y Neurocirugía Manuel Velasco Suárez, Mexico City 14269, Mexico; draztrujillo@gmail.com; 6 Clinical Research Laboratory, Instituto Nacional de Neurología y Neurocirugía Manuel Velasco Suárez, Mexico City 14269, Mexico; bollneur@gmail.com; 7 Geriatrics Service, Hospital Regional “Lic. Adolfo López Mateos”, Instituto de Seguridad y Servicios Sociales de los Trabajadores del Estado, Mexico City 01030, Mexico; rosaliasola@gmail.com; 8 Department of Biological Systems, Universidad Autónoma Metropolitana Unidad Xochimilco, Mexico City 04960, Mexico

**Keywords:** Alzheimer’s disease, pharmacogenetics, *ACHE*, *BCHE*, donepezil, memantine

## Abstract

**Background/Objectives**: Several genetic factors are related to the risk of Alzheimer’s disease (AD) and the response to cholinesterase inhibitors (ChEIs) (donepezil, galantamine, and rivastigmine) or memantine. However, findings have been controversial, and, to the best of our knowledge, admixed populations have not been previously evaluated. We aimed to determine the impact of genetic and non-genetic factors on the risk of AD and the short-term response to ChEIs and memantine in patients with AD from Mexico. **Methods**: This study included 117 patients from two specialty hospitals in Mexico City, Mexico. We evaluated cognitive performance via clinical evaluations and neuropsychological tests. Nineteen variants in *ABCB1*, *ACHE*, *APOE*, *BCHE*, *CHAT*, *CYP2D6*, *CYP3A5*, *CHRNA7*, *NR1I2*, and *POR* were assessed through TaqMan assays or PCR. **Results**: Minor alleles of the *ABCB1* rs1045642, *ACHE* rs17884589, and *CHAT* rs2177370 and rs3793790 variants were associated with the risk of AD; meanwhile, *CHRNA7* rs6494223 and *CYP3A5* rs776746 were identified as low-risk variants in AD. *BCHE* rs1803274 was associated with worse cognitive functioning. None of the genetic and non-genetic factors studied were associated with the response to pharmacological treatment. **Conclusions**: We identified potential genetic variants related to the risk of AD; meanwhile, no factor was observed to impact the response to pharmacological therapy in patients with AD from Mexico.

## 1. Introduction

Alzheimer’s disease (AD) and other dementias are a global health challenge, with a notable prevalence, cost, and impact [1]. AD is the most frequent cause of dementia in individuals aged 65 years or older, accounting for an estimated 60–80% of cases [2]. According to the World Alzheimer Report 2021, over 55 million persons live with dementia worldwide, and this figure is projected to reach 78 million by 2030 [3].

AD is characterized by cognitive impairment, progressive neurodegeneration, the formation of plaques containing amyloid β-peptide, and neurofibrillary tangles composed of hyperphosphorylated tau [4]. At disease onset, patients with AD present deficits in recent (short-term) memory, word finding, and language abilities. The disease gradually progresses to global cognitive impairment, which can be accompanied by a variety of abnormal neurological and neuropsychiatric symptoms [5,6].

The current treatment options for AD comprise non-pharmacological and pharmacological strategies, and it is highly recommended that patients receive both. Non-pharmacological interventions consist of methods focused on cognitive stimulation, such as holistic techniques (i.e., reality orientation, cognitive stimulation therapy, and reminiscence therapy), brief psychotherapy, cognitive methods (i.e., spaced retrieval), and alternative strategies (i.e., music therapy and bright light therapy) [7,8].

Drugs for AD remain limited; since 1998, there have been more than 100 attempts to develop an effective drug to treat the disease, but only four have been approved [9]. The pharmacological strategies available for the treatment of the cognitive and behavioral symptoms of AD target the neurotransmitter systems related to the course of the disease. Cholinesterase inhibitors (ChEIs) include donepezil (DPZ), galantamine (GAL), and rivastigmine (RIV). These drugs bind to and inhibit acetylcholinesterase and butyrylcholinesterase to increase synaptic acetylcholine levels and improve cholinergic neurotransmission in the hippocampus. Meanwhile, the N-methyl-D-aspartate antagonist (NMDA) memantine (MEM) blocks the effects of the pathologically elevated tonic levels of glutamate, which is cytotoxic and is a crucial factor contributing to the neuronal loss and cell death observed in AD [10].

In addition to the lack of choices in pharmacological treatment, a low rate of good clinical responses (27.8%) has been reported for ChEIs [11]. Genetic and non-genetic factors could partly explain this variability in drug response. In this sense, pharmacogenetics can potentially improve the discovery, development, and use of medicines through the study of the influence of genetic variants on the clinical outcome of several diseases, including AD [12,13]. Non-genetic factors (i.e., co-treatment, co-morbidities, and disease severity) can also significantly impact the drug response in AD. Some reports have identified age, gender, and dosage as predictors of the outcome of the ChEI [14,15,16]; however, other studies have not stated factors affecting the response to ChEIs or MEM [17,18].

In the scientific literature, there are, to the best of our knowledge, more than 30 pharmacogenetic studies on AD, mainly including Caucasian patients, although other populations have been included (i.e., Korean, Brazilian, and Indian populations). Several genetic biomarkers investigated in these studies are related to the metabolism (i.e., *CYP2D6*, *CYP3A4*, *CYP3A5*, *NR1I2*, and *ABCB1*) and/or the mechanism of action (i.e., *APOE*, *ACHE*, *BCHE*, and *CHAT*) of ChEIs, as well as MEM. However, conclusions have been controversial, and an interethnic variability among the association results is markedly observed [13]. To the best of our knowledge, no studies to date have evaluated the inter-individual variability in AD treatment response in admixed populations, such as Mexican Mestizo (MM) populations, who present a particular genetic background that could contribute to the knowledge of the pharmacogenetics and variations in the risk of AD and its treatment. Therefore, we aimed to determine the impact of genetic and non-genetic factors on the risk of AD and the short-term response to ChEIs and MEM.

## 2. Materials and Methods

### 2.1. Subjects

We enrolled a total of 117 patients (with an age range of 40–90 years) with a diagnosis of AD (mild or moderate) from the Genetics Department of the Instituto Nacional de Neurología y Neurocirugía (INNN) and the Geriatric Service of the Hospital Regional Adolfo López Mateos (HRALM), in Mexico City, Mexico. The patients’ clinical and demographic data are presented in Table 1. The diagnosis of AD was established according to the criteria of the National Institute of Neurological and Communicative Disorders and the Alzheimer’s Disease and Related Disorders Association (NINCDS-ADRDA 2007) [19,20,21]. After diagnosis, the patients were prescribed a ChEI and/or MEM. This study was conducted according to the Declaration of Helsinki (2024) and approved by the local Ethics Committees (38/16 INNN and #195.2017 HRALM). Written informed consent was obtained from all of the participants’ primary caregivers and volunteers.

The frequencies of genetic variants not previously reported among MM populations (*ACHE*, *BCHE*, *CHAT*, *CHRNA7*, and *POR*) were determined in DNA samples that were taken from 300 unrelated healthy volunteers (mean age: 26.9 ± 10.7 years; 193 were female) of MM ethnic origin and stored in the laboratory biobank. The subjects originated from the Mexico Central Area, which comprises Mexico City and the states of Mexico, Tlaxcala, Hidalgo, Morelos, and Puebla.

In this study, we only included subjects with both parents and four grandparents, both maternal and paternal, of MM origin, thus ensuring ethnic homogeneity among all the participants.

### 2.2. DNA Extraction and Genotyping

All the participants provided a 10 mL sample of peripheral blood, which was collected in tubes with acid citrate dextrose and employed for the isolation of genomic DNA using standard techniques. The DNA samples were quantified, verified for purity, and stored at 4 °C until use.

The pharmacogenes relevant to the AD treatment studied were *ABCB1*, *ACHE*, *APOE*, *BCHE*, *CHAT*, *CYP2D6*, *CYP3A5*, *CHRNA7*, *NR1I2*, and *POR*. The genetic variants of *ABCB1*, *ACHE*, *APOE*, *BCHE*, *CHAT*, *CYP3A5*, *CHRNA7*, *NR1I2*, and *POR* were determined using TaqMan^®^ SNP Genotyping assays (Table 2) in a Step One Plus™ Real-Time PCR system (Applied Biosystems™, Carlsbad, CA, USA) according to the supplier’s methodology.

*APOE* genotyping consisted of the use of the restriction enzyme isoform method reported by Hixson and Vernier, with slight modifications [22]. *CYP2D6* variants were determined using previously described PCR-RFLP and Real-Time PCR methods: CYP2D6*2 [23]; *3, *4, and gene multiplication (×N) [24]; and *5, *6, *10, and *17 [25]. The relationship between the *CYP2D6* genotype and the predicted metabolizer status was evaluated using the “activity score” [26,27]. The value assigned to the reference alleles CYP2D6 *1 and *2 was 1; that assigned to CYP2D6*3, *4, *4 × N, *5, and *6 was 0; that assigned to CYP2D6*10 and *17 was 0.5; and that assigned to multiplications of active *CYP2D6* (*1 × N or *2 × N) was *n* (the number of copies). The subjects with a value of 0 or 0.5 and more than two *CYP2D6* active genes were classified as PMs and UMs, respectively, and the remaining subjects were classified as EMs.

*APOE* genotyping was performed in all patients. Meanwhile, the patients’ treatment was considered for the selection of the pharmacogenetic variants due to differences in the pharmacokinetics and pharmacodynamics of the studied drugs. Thus, the patients receiving DPZ treatment (*n* = 44) were genotyped for the *CYP2D6*, *ABCB1*, *ACHE*, *BCHE*, *CHAT*, *CYP3A5*, *NR1I2*, *CHRNA7*, and *POR* variants. The patients treated with GAL (*n* = 17) were genotyped for the *CYP2D6*, *ABCB1*, *ACHE*, *BCHE*, *CHAT*, *CYP3A5*, *CHRNA7*, and *POR* variants. In the patients taking RIV (*n* = 10), the variants of *CYP2D6*, *ABCB1*, *ACHE*, *BCHE*, *CHAT*, and *CHRNA7* were assessed, and the *NR1I2* and *CHRNA7* variants were assessed in the patients treated with MEM.

### 2.3. Drug Response Assessment

Neuropsychological tests were conducted only in the patients with AD for the assessment of the short-term response to the drugs used in this study. These tests comprised the Mini-Mental State Examination (MMSE); Clock Drawing Test (CLOCK); Semantic Verbal Fluency test (SVF); Phonological Verbal Fluency test (PVF); Katz Index; Global Deterioration Scale (GDS); and Lawton–Brody Scale. All of these have been previously employed in a Mexican population [28,29,30] and were applied by neuropsychologists trained in examining cognitive performance. Evaluations were performed twice during the study: prior to the onset of the treatment (Time 1) and 6 months later (Time 2). Responders were patients who obtained the same or better scores in the neuropsychological tests after 6 months of treatment with a ChEI and/or MEM, and non-responders were those who demonstrated worsened cognitive performance according to the test scores.

Adverse drug reactions (ADRs) to the ChEIs and/or MEM were elicited from the patients’ primary caregivers during the clinical examination.

### 2.4. Statistical Analysis

The categorical variables are presented as the frequency and percentage values. The continuous variables are reported as the mean and standard deviation (mean ± sd) values or the median [Interquartile range, IQR] for non-normally distributed data. The Shapiro–Wilk and Kolmogorov–Smirnov tests were employed to assess normal distributions. The allele and genotype frequencies among the groups were compared using the Fisher exact test with Bonferroni correction for multiple comparisons in PLINK v1.07 statistical software [31]. The association of categorical non-genetic variables with the treatment response was evaluated using the Fisher exact test, and continuous data were compared between the responders and non-responders using the Mann–Whitney U or Kruskal–Wallis test. The differences in the MMSE scores between Times 1 and 2 were evaluated using the Wilcoxon Rank test. A *p*-value of <0.05 was considered statistically significant, and the tests were performed using RStudio v. 1.3.1073 [32].

## 3. Results

### 3.1. Clinical and Demographic Data in Responder and Non-Responder Groups

In this study, the sample of MM patients comprised 74 females and 43 males, and more than half presented familial and early-onset AD. The majority of these patients (69.20%) presented depression, and hypertension and diabetes mellitus were also identified. In total, 80 patients were treated with only one ChEI or MEM, while 37 received combined treatment with one ChEI + MEM. In addition, some patients were treated with antidepressants and antipsychotics. According to the neuropsychological tests, nearly 50% of the patients responded to the AD treatment, and only 16 reported ADRs (Table 1).

The neuropsychological test scores revealed that the patients initially presented mild-to-moderate AD, and, as a whole, a decrease in these scores was observed at Time 2 (Table 1). According to the Katz Index of Independence in Activities of Daily Living (ADL), at treatment onset (Time 1), 59% of the patients were classified as grade A or B (independent or independent in all basic activities of daily life except for one); 35.9% were classified as grade C or D (dependent for bathing and other essential activities or dependent for bathing, dressing, and other essential activities); and 5.1% were classified as grade E, F, or G (dependent for bathing, dressing, toileting, and/or transferring or dependent for the six essential activities, namely bathing, dressing, toileting, transferring, continence, and feeding). At Time 2, 51.3% of the patients were classified as grade A or B, 35.9% were classified as grade C or D, and 12.8% were classified as grade E, F, or G.

Table 3 shows the non-genetic factors of the responder and non-responder groups. The non-responders were slightly younger, and their disease onset was earlier than the responders, although this was not statistically significant. The years of scholarship, the proportion of females, and the frequency of comorbidities were also similar among the groups. Thus, none of the included non-genetic variables (age, onset age, scholarship, sex, AD type, co-treatment, or comorbidities) were observed to impact the response to the ChEIs or MEM.

Regarding cognitive performance, the MMSE, SVF, PVF, and CLOCK scores were similar between the groups at Time 1; contrariwise, there were significant differences among the GDS, KATZ, and Lawton–Brody results. As expected, all scores at Time 2 differed between the responders and non-responders, with better performance in the former group.

### 3.2. Study of Genetic Variants in Patients with Alzheimer’s Disease and Cognitive Performance

The frequencies of the genetic variants included in this study in the healthy volunteers and patients are presented in Supplementary Materials S1 and S2. Our research group previously reported the frequencies of the *ABCB1* [33], *CYP3A5*, and *NR1I2* [34] variants in controls. The genotypic frequencies were found to be in agreement with the Hardy–Weinberg equilibrium in all cases (*p* > 0.05) and were similar to those reported for Mexican individuals from Los Angeles, California, and other populations worldwide [35]. Nevertheless, some minor allele frequencies were significantly different when compared between patients with AD and MM healthy volunteers (Table 4). Minor alleles of the *ABCB1* rs1045642, *ACHE* rs17884589, and *CHAT* rs2177370 and rs3793790 variants were identified as risk factors for AD; meanwhile, *CHRNA7* rs6494223 and *CYP3A5* rs776746 were identified as low-risk variants in AD. However, after Bonferroni correction for multiple comparisons, only *ACHE* rs17884589 and *CHRNA7* rs6494223 showed an association (p_c_ = 0.019 and 0.006, respectively).

**Figure 1:**
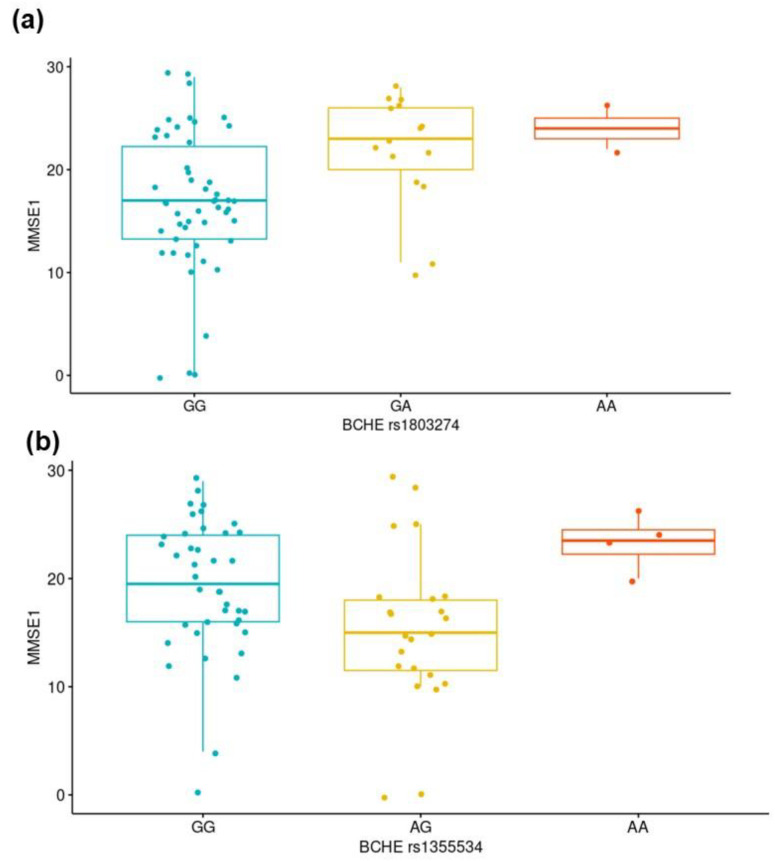
Differences in the Mini-Mental State Examination-Time 1 (MMSE1) scores according to *BCHE* (butyrylcholinesterase gene) (**a**) rs1803274 (Kruskal–Wallis test, *p* = 0.012) and (**b**) rs1355534 (Kruskal–Wallis test, *p* = 0.014) genotypes.

We sought to determine whether the genetic variants studied could impact the cognitive performance observed in the patients before they started their pharmacological AD treatment. Therefore, we compared the MMSE1 scores according to the genotypes of genes coding for brain-related proteins (*APOE*, *ABCB1*, *ACHE*, *BCHE*, *CHAT*, and *CHRNA7*). We found lower MMSE1 scores among the patients carrying the CC genotype of *BCHE* rs1803274 than among those carrying the CT genotype (*p* = 0.012; Figure 1a), suggesting worse cognitive impairment. The MMSE1 scores also differed when the genotypes of the *BCHE* rs1355534 variant were considered (*p* = 0.014; Figure 1b). The MMSE1 score did not differ among the remaining studied variants (Supplementary Material S3).

**Figure 2:**
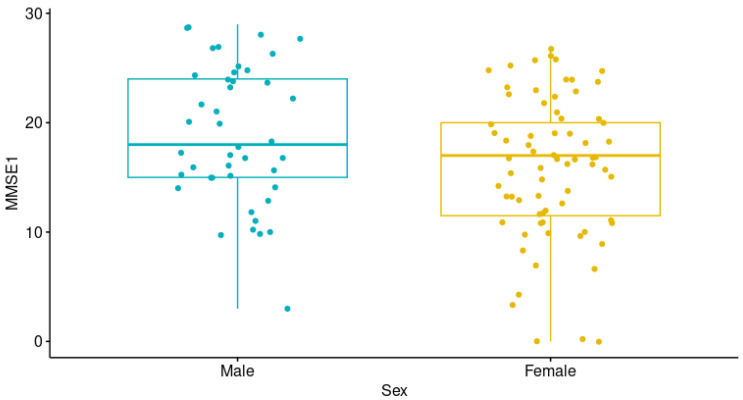
Differences in the Mini-Mental State Examination-Time 1 (MMSE1) scores according to sex (Mann–Whitney U test, *p* = 0.034).

Then, we sought to determine whether any of the non-genetic factors affected the cognitive performance of the patients at the start of the study, that is, the MMSE1 score. We found that sex and scholarship were related to the MMSE1 score, as males exhibited higher scores than females (*p* = 0.034; Figure 2), while a weak positive correlation was observed between years of scholarship and the first MMSE score (*p* = 0.002; rho = 0.282). Age, AD type, age at AD onset, years of AD evolution, late/early AD onset, and co-morbidities (depression, systemic arterial hypertension, and type 2 diabetes mellitus [T2DM]) were not related to the MMSE1 score (Supplementary Material S4).

**Figure 3:**
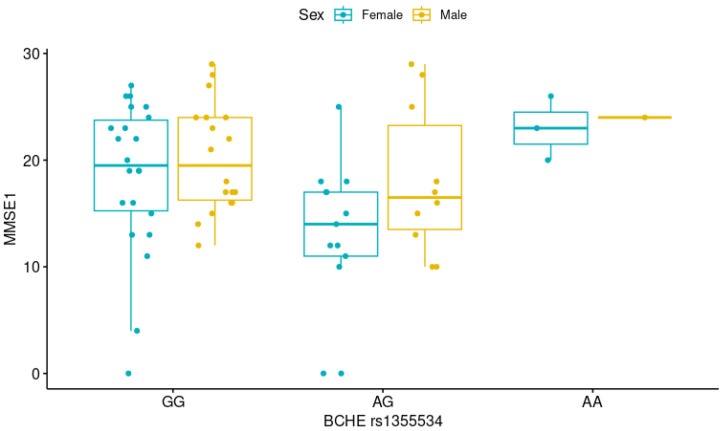
Differences in Mini-Mental State Examination-Time 1 (MMSE1) score according to *BCHE* (butyrylcholinesterase gene) rs1355534 variants and sex (GG *n* = 40, AG *n* = 23, AA *n* = 4). The MMSE1 score differed between males and females with the AG genotype (*p* = 0.017, generalized linear model).

Therefore, we used a generalized linear model to evaluate the association between the *BCHE* variants and the MMSE1 score, adjusting for sex. We observed a significant difference in the MMSE1 scores among individuals with the AG rs1355534 genotype when sex was considered (*p* = 0.017), with females with the AG genotype demonstrating lower performance in the MMSE1 test than AG males (Figure 3).

### 3.3. Study of Pharmacogenetic Variants with the Short-Term Response to Donepezil, Galantamine, Rivastigmine, and Memantine

We examined whether the frequencies of the genetic variants included in this study differed between the groups of responders and non-responders to donepezil, galantamine, rivastigmine, and memantine. None of the pharmacogenetic variants were associated with the treatment response (Table 5), even after adjusting for sex, a relevant co-variable impacting cognitive performance according to the results above mentioned. A lack of association was also found in the analyses of the CYP2D6-predicted phenotypes (Supplementary Material S5). These results should be taken with caution, as the subclassification of groups according to the primary treatment led to some subgroups containing fewer than 10 subjects, thus decreasing the statistical power of partial studies. Further studies are warranted to rule out the influence of pharmacogenetic variants in the treatment of AD.

## 4. Discussion

Alzheimer’s disease (AD) has become a global health concern due to its rapid increase in prevalence worldwide. Knowledge related to the pathogenesis and treatment response in patients with AD could contribute to improving the diagnosis and therapy of the disease. Herein, we evaluated the risk of AD in relation to variants in neurotransmitter genes. To the best of our knowledge, this is the first report to show an association between the *BCHE* rs1803274 variant and the MMSE1 score in untreated patients with AD.

Several risk factors have been related to AD; for instance, a recent study reported the impact of high fasting blood glucose levels, a high body mass index, and smoking in susceptibility to dementia [36]. In addition, several genetic variants have been associated with AD, such as *APOE*, *APP*, *PSEN1*, and *PSEN2* [37], as well as other variants recently reported in immunological pathways [38].

We observed a probable association between *ABCB1* rs1045642, *ACHE* rs17884589, *CHAT* rs2177370 and rs3793790, and *CHRNA7* rs6494223 and the differential risk for AD. The efflux transporter ABCB1 is related to beta-amyloid clearance in the brain [39]. Indeed, the rs1045642 variant was found to be associated with susceptibility to AD in a meta-analysis [40]. Moreover, in accordance with our study, a recent investigation reported that the rs1045642 variant was associated with the risk of AD, but it did not report that it was associated with the response to donepezil [41]. In previous studies, the *CHAT* rs2177370 and rs3793790 variants were evaluated and found to be associated with the response to acetylcholinesterase inhibitors [42,43] but not AD susceptibility. However, another *CHAT* variant (rs3810950) was reported to be associated with AD risk in a Czech population [44]. Likewise, previous studies investigated the relationship of the *CHRNA7* rs6494223 variant with the response to AD treatment [45,46] but not its association with susceptibility to the disease. This variant was the only one that remained significant after correction for multiple comparisons. It is an intronic variant in the α7 nicotinic acetyl-choline receptor gene that has been related to neurological diseases, such as bipolar disorder [47,48], and delusional symptoms in AD [49], probably due to instability in the receptor’s expression [49]. It is known that this receptor is involved in the development of dementia due to the increase in cholinergic neurotransmission, the induction of long-term potentiation, and its neuroprotective effects [50]. However, further studies are warranted to explain the involvement of this *CHRNA7* variant in the risk of AD and other dementias.

We also observed a difference in the frequency of the *CYP3A5* rs776746 variant among the patients with AD and healthy volunteers, as the minor allele was associated with a low risk of AD. CYP3A5 has been found in the brain [51], and *CYP3A5* genetic variants have previously been associated with schizophrenia, which could support the involvement of the enzyme in the neurobiological process. Notwithstanding this, these interesting results require further analysis to elucidate the participation of this enzyme in neurological disorders such as AD.

The MMSE baseline score differed according to the genotype of the studied *BCHE* variants (rs1803274 and rs1355534), which could indicate alterations in enzymatic activities due to the genetic variant and its impact on the cognitive process; however, further studies are warranted to elucidate the precise mechanism. Nevertheless, the involvement of BChE in cognition has been widely described; for instance, a recent study reported an association between the inhibition of BChE due to exposure to a pesticide (chlorpyrifos) and the MMSE score in farmers [52].

Herein, none of the genetic or non-genetic factors were observed to affect the AD treatment. Several pharmacogenetic studies have found controversial results regarding the association of genetic variants with the response to DPZ, GAL, RIV, and/or MEM [13]. A recent study evaluated the pharmacogenetics of donepezil and memantine in healthy subjects and did not find an association between any of the included genetic variants and the pharmacokinetic parameters of these drugs [53]. Meanwhile, in another report, the CYP2D6 phenotype extrapolated from the genotype was found to be related to DPZ plasma concentrations in Thai patients with AD [54], thereby contributing to the controversy in this field. Therefore, the potential benefits of pharmacogenetics in AD remain unknown, and the search for further biomarkers in different pathways is still necessary.

A major limitation of our study is the small sample size, which certainly underpowers the association results. Notwithstanding this, the rigorous clinical actions required in the follow-up of the patients precluded the inclusion of a larger sample size. In addition, conducting neuropsychological tests in healthy elderly individuals would be valuable for examining the association between MMSE scores and the *BCHE* variant.

Nevertheless, we demonstrated the probable participation of *ABCB1*, *ACHE*, *CHAT*, *CHRNA7*, and *CYP3A5* in the risk of AD, and we found that *BCHE* rs1803274 impacts the MMSE1 score among patients with AD. None of the studied variants were found to be related to the patients’ response to AD treatment; however, further studies are still warranted to identify pharmacogenomic biomarkers in this disease, as well as studies evaluating the long-term response. This study contributes to the knowledge on AD and forms a basis for the design of future investigations of the disease.

## Figures and Tables

**Table 1:** Clinical and sociodemographic data of the patients with Alzheimer’s disease included in this study (*n* = 117).

Variable	Median (Interquartile Range (IQR))
Age (years)	64 (56–74)
Age at onset (years)	59 (51–69)
Years of disease evolution	5 (3–7)
Years of education	9 (5–14)
Neuropsychological tests	
Time 1 ^a^	
MMSE-1	17 (12–23)
SVF-1	7 (4–11)
PVF-1	3 (1–7)
CLOCK-1	0 (0–2)
GDS-1	4 (3–4)
Lawton Brody-1	4 (0–4)
Time 2 ^b^	
MMSE-2	15 (10–20)
SVF-2	6 (3–10)
PVF-2	3 (0–6)
CLOCK-2	0 [0–1]
GDS-2	4 [3–5]
Lawton Brody-2	2 [0–4]
Gender male/female *n* (%)	43 (36.8)/74 (63.2)
AD sporadic/familial	49 (41.90)/68 (58.10)
AD early/late onset	72 (61.50)/45 (38.50)
Co-morbidities	
Depression	81 (69.20)
Hypertension	39 (33.30)
Diabetes mellitus	18 (15.40)
Treatment	
Monotherapy (DPZ, GAL, RIV, or MEM)	80 (68.40)
DPZ + MEM	20 (17.10)
GAL + MEM	12 (10.30)
RIV + MEM	5 (4.30)
Co-treatment	
Antidepressant	81 (69.20)
Antipsychotic	32 (27.40)
Responders/non-responders	58 (49.60)/59 (50.40)
ADR/no ADR	16 (13.70)/101 (86.30)

^a^ Onset of treatment; ^b^ 6 months later. AD, Alzheimer’s disease; ADR, adverse drug reaction; CLOCK, Clock Drawing Test; DPZ, donepezil; GAL, galantamine; GDS, Global Deterioration Scale; MEM, memantine; MMSE, Mini-Mental State Examination; PVF, Phonological Verbal Fluency test; RIV, rivastigmine; SVF, Semantic Verbal Fluency test.

**Table 2:** Genetic variants evaluated in this study.

Gene	NCBI Reference	TaqMan Assay
*ABCB1*	rs1128503	C___7586662_10
	rs2032582	C_11711720C_30 C_11711720D_40
	rs1045642	C__7586657_20
*ACHE*	rs1799806	C_27168953_30
	rs17884589	C_34446515_10
	rs10953305	C_2607820_20
*BCHE*	rs1803274	C_27479669_20
	rs1355534	C_8834703_20
*CHAT*	rs2177370	C_224405_10
	rs3793790	C_122323_20
*CYP3A5*	rs776746 (CYP3A5*3)	C_26201809_30
	rs10264272 (CYP3A5*6)	C__30203950_10
*NR1I2*	rs2461817	C__15803606_20
	rs7643645	C___1834250_10
	rs3814055	C__27504984_30
	rs2276707	C__15882324_10
	rs3814058	C__11231740_10
*CHRNA7*	rs6494223	C___1483016_10
*POR*	rs1057868	C___8890131_30

**Table 3:** Clinical and demographical variables in patients with Alzheimer’s disease classified according to the response to pharmacological therapy (*n* = 117).

Variable	Non-Responders *n* = 58 (%)	Responders *n* = 59 (%)	*p*-Value
Age, yrs	61.5 ([55–71.5)	67 (57–76)	0.081
Onset age, yrs	56 (50.3–66.8)	60 (53.5–69.5)	0.148
Scholarship, yrs	9 (6–14)	9 (4–15)	0.363
Sex			
Male	20 (34.5)	23 (39.0)	0.702
Female	38 (65.5)	36 (61.0)	
AD type			
Sporadic	20 (34.5)	29 (49.1)	0.135
Familial	38 (65.5)	30 (50.8)	
Onset AD			
Early	38 (65.5)	34 (57.6)	0.449
Late	20 (34.5)	25 (42.4)	
Co-treatment			0.333
Monotherapy	36 (62.1)	43 (72.9)	0.240
DPZ + MEM	13 (22.4)	7 (11.9)	0.148
GAL + MEM	5 (8.6)	7 (11.9)	0.762
RIV + MEM	4 (6.9)	2 (3.4)	0.439
Comorbidities			
Depression	40 (69.0)	42 (71.2)	0.842
SAH	16 (27.6)	23 (39.0)	0.240
T2DM	7 (12.1)	11 (18.6)	0.443
Antidepressant			
Citalopram	20 (34.5)	24 (40.7)	0.514
Escitalopram	5 (8.6)	9 (1.5)	
None	20 (34.5)	16 (27.1)	
Other	13 (22.4)	10 (16.9)	
Antipsychotic	19 (32.8)	13 (22.0)	0.218
Neuropsychological tests			
MMSE1	17 (12–22)	17 (13–22)	0.511
SVF1	7 (3–10)	7 (4–12)	0.494
PVF1	3 (1–6)	4 (1–8.5)	0.180
CLOCK1	0 (0–1)	0 (0–2)	0.283
GDS1	4 (3–5)	3 (3–4)	0.008
Lawton–Brody 1	2 (0–4)	4 (2–6)	0.005
KATZ1			
A-B	27 (46.5)	42 (71.2)	0.022
C-D	27 (46.5)	15 (25.4)	
E-F	4 (7.0)	2 (3.4)	
MMSE2	10 (7–16)	20 (13–24)	<0.001
SVF2	4 (0–7)	7 (4–12)	<0.001
PVF2	1 (0–3)	4 (1–8)	0.001
CLOCK2	0 (0–0)	0 (0–1)	0.003
GDS2	4 (4–5)	3 (3–4)	<0.001
Lawton–Brody 2	0 (0–2)	3 (2–6)	<0.001
KATZ2			
A-B	20 (34.5)	40 (67.8)	0.001
C-D	27 (46.5)	14 (23.7)	
E-F	11 (19.0)	5 (8.5)	

AD, Alzheimer’s disease; CLOCK, Clock Drawing Test; DPZ, donepezil; GAL, galantamine; GDS, Global Deterioration Scale; MEM, memantine; MMSE, Mini-Mental State Examination; PVF, Phonological Verbal Fluency test; RIV, rivastigmine; SAH, systemic arterial hypertension; T2DM, type 2 diabetes mellitus; SVF, Semantic Verbal Fluency test.

**Table 4:** Minor allele frequencies between patients with Alzheimer’s disease and healthy volunteers from Mexico.

Gene	Variant	MAF/AD	MAF/MM	Reference	*p* Value	OR (95% CI)
*APOE*	rs7412 (ε4)	0.106	0.085	[36]	0.523	-
*ABCB1*	rs1128503	0.492	0.49	[33]	0.985	-
	rs1045642	0.613	0.49		0.014	1.34 (1.10–2.43)
	rs2032582 (A/T)	0.040/0.395	0.07/0.42		0.221	-
*ACHE*	rs1799806	0.239	0.172	Present work	0.07	-
	rs17884589	0.261	0.147		0.001	2.08 (1.33–3.25)
	rs10953305	0.194	0.243		0.223	-
*BCHE*	rs1803274	0.142	0.11	Present work	0.298	-
	rs1355534	0.231	0.312		0.066	-
*CHAT*	rs2177370	0.397	0.285	Present work	0.01	1.65 (1.12–2.43)
	rs3793790	0.213	0.140		0.032	1.66 (1.04–2.66)
*CHRNA7*	rs6494223	0.333	0.471	Present work	<0.001	0.56 (0.41–0.77)
*POR*	rs1057868	0.275	0.259	Present work	0.704	-
*CYP3A5*	rs776746	0.135	0.263	[34]	0.003	0.44 (0.25–0.77)
	rs10264272	0.025	0.005		0.094	-
*NR1I2*	rs2461817	0.391	0.348	[34]	0.288	-
	rs7643645	0.446	0.387		0.153	-
	rs2276707	0.141	0.175		0.284	-
	rs3814055	0.429	0.407		0.398	-
	rs3814058	0.141	0.175		0.284	-

AD, patients with Alzheimer’s disease; CI, confidence interval; MAF, minor allele frequency; MM, Mexican Mestizo healthy volunteers; OR, odds ratio.

**Table 5:** Study of association between pharmacogenetic variants and the short-term response to donepezil, galantamine, rivastigmine, and memantine among patients with Alzheimer’s disease.

Genetic Variant (Allele/Genotype)	MAF Non-Responders	MAF Responders	*p* Value	*p* Value Adjusted for Sex
Donepezil	*n* = 22	*n* = 22		
*APOE* rs7412 (ε4)	0.068	0.105	0.839	0.768
*ABCB1*				
rs1128503	0.500	0.500	1.000	0.969
rs1045642	0.476	0.316	0.144	0.185
rs2032582 (A/T)	0.500	0.395	0.345	0.420
*ACHE*				
rs1799806	0.273	0.159	0.195	0.992
rs17884589	0.182	0.250	0.437	0.769
rs10953305	0.295	0.136	0.070	0.999
*BCHE*				
rs1803274	0.114	0.250	0.097	0.680
rs1355534	0.227	0.182	0.597	0.844
*CHAT*				
rs2177370	0.341	0.409	0.509	0.391
rs3793790	0.227	0.182	0.597	0.900
*CHRNA7*				
rs6494223	0.318	0.386	0.503	0.161
*POR*				
rs1057868	0.318	0.159	0.080	0.513
*CYP3A5*				
rs776746	0.114	0.159	0.534	0.484
rs10264272	0.023	0.045	0.557	0.541
*NR1I2*				
rs2461817	0.341	0.454	0.276	0.488
rs7643645	0.432	0.545	0.286	0.529
rs3814055	0.386	0.523	0.199	0.301
rs2276707	0.091	0.114	0.725	0.767
rs3814058	0.091	0.114	0.725	0.767
Galantamine	*n* = 9	*n* = 8		
*APOE* rs7412 (ε4)	0.062	0.250	0.330	0.362
*ABCB1*				
rs1128503	0.500	0.500	1.000	0.566
rs1045642	0.357	0.429	0.144	0.699
rs2032582 (A/T)	0.357	0.429	0.699	0.420
*ACHE*				
rs1799806	0.143	0.437	0.079	0.286
rs17884589	0.429	0.312	0.510	0.304
rs10953305	0.000	0.125	0.171	0.999
*BCHE*				
rs1803274	0.071	0.000	0.277	NA
rs1355534	0.286	0.437	0.389	0.836
*CHAT*				
rs2177370	0.437	0.437	1.000	1.000
rs3793790	0.125	0.187	0.626	0.714
*CHRNA7*				
rs6494223	0.312	0.437	0.645	0.871
*POR*				
rs1057868	0.312	0.437	0.465	0.871
*CYP3A5*				
rs776746	0.143	0.125	0.886	0.906
rs10264272	0.000	0.000	NA	NA
Rivastigmine	*n* = 5	*n* = 3		
*APOE* rs7412 (ε4)	0.200	0.000	0.696	NA
*ABCB1*				
rs1128503	0.500	0.333	0.492	1.000
rs1045642	0.429	0.167	0.260	0.618
rs2032582 (A/T)	0.400	0.167	0.699	0.676
*ACHE*				
rs1799806	0.214	0.167	0.807	0.718
rs17884589	0.286	0.167	0.573	0.987
rs10953305	0.286	0.167	0.573	1.000
*BCHE*				
rs1803274	0.214	0.167	0.807	0.811
rs1355534	0.071	0.167	0.515	0.547
*CHAT*				
rs2177370	0.429	0.333	0.690	0.997
rs3793790	0.357	0.167	0.394	0.934
*CHRNA7*				
rs6494223	0.312	0.437	0.645	0.871
Memantine	*n* = 22	*n* = 26		
*CHRNA7*				
rs6494223	0.319	0.337	0.813	0.731
*NR1I2*				
rs2461817	0.419	0.370	0.517	0.731
rs7643645	0.419	0.402	0.827	0.371
rs3814055	0.351	0.413	0.417	0.480
rs2276707	0.122	0.174	0.349	0.955
rs3814058	0.122	0.174	0.349	0.955

MAF, minor allele frequency; NA, not applicable.

## Data Availability

Data are available to interested researchers upon request.

## Supplementary Materials

The following supporting information can be downloaded at https://www.mdpi.com/article/10.3390/neurolint17050065/s1: Table S1: Allele and genotype frequencies of genetic variants included in this study and not previously reported in Mexican Mestizo individuals (*n* = 300); Table S2: Allele and genotype frequencies of genetic variants in Mexican Mestizo individuals with Alzheimer’s disease; Table S3: Evaluation of ABCB1, ACHE, BCHE, CHAT, and CHRNA7 genetic variants with regard to MMSE1 score; Table S4: Evaluation of non-genetic variables with regard to the first MMSE score; Table S5: Association between CYP2D6-predicted phenotype and the response to donepezil, galantamine, and rivastigmine among patients with Alzheimer’s disease.

## Abbreviations

The following abbreviations are used in this manuscript:

AD	Alzheimer’s disease
ChEI	Cholinesterase inhibitor
DPZ	Donepezil
GAL	Galantamine
RIV	Rivastigmine
NMDA	N-methyl-D-aspartate antagonist
*CYP2D6*	Cytochrome P450 family 2 subfamily D member 6
*CYP3A4*	Cytochrome P450 family 3 subfamily A member 4
*CYP3A5*	Cytochrome P450 family 3 subfamily A member 5
*NR1I2*	Nuclear receptor subfamily 1 Group I member 2
*ABCB1*	ATP binding cassette subfamily B member 1
*APOE*	Apolipoprotein E
*ACHE*	Acetylcholinesterase
*BCHE*	Butyrylcholinesterase
*CHAT*	Choline O-acetyltransferase
MEM	Memantine
MM	Mexican Mestizo
INNN	Instituto Nacional de Neurología y Neurocirugía
HRALM	Hospital Regional Adolfo López Mateos
*CHRNA7*	Cholinergic receptor nicotinic alpha 7 subunit
*POR*	Cytochrome P450 oxidoreductase
MMSE	Mini-Mental State Examination
GDS	Global Deterioration Scale
PVF	Phonological Verbal Fluency test
SVF	Semantic Verbal Fluency test
ADRs	Adverse drug reactions
ADL	Activities of daily living
SAH	Systemic arterial hypertension
T2DM	Type 2 diabetes mellitus
MAF	Minor allele frequency
CI	Confidence interval
OR	Odds ratio
