# Supplementary material for: Variants in Neurotransmitter-Related Genes Are Associated with Alzheimer’s Disease Risk and Cognitive Functioning but Not Short-Term Treatment Response
Source: Neurol Int. 2025 Apr 24;17(5):65. doi: 10.3390/neurolint17050065 (PMC12114220; doi:10.3390/neurolint17050065)
Supplement: Supplementary file 1 [file neurolint-17-00065-s001.zip › neurolint-3567150-supplementary.pdf]

Table S1. Allele and genotype frequencies of genetic variants included in the study, non previously reported in Mexican Mestizos (n=300).

Gene	Variant	Allele frequency (95% CI)	Genotype frequency
ACHE	rs1799806<br>g.9884C>G,<br>p.Pro592Arg	C = 0.172 (0.14-0.20)<br>G = 0.828 (0.80-0.86)	CC = 0.030<br>CG = 0.283<br>GG = 0.687
	rs17884589<br>g.4831C>T	C = 0.853 (0.82-0.88)<br>T = 0.147 (0.12-0.18)	CC = 0.733<br>CT = 0.240<br>TT = 0.027
	rs10953305<br>g.5974C>T	C = 0.243 (0.21-0.28)<br>T = 0.757 (0.72-0.79)	CC = 0.073<br>CT = 0.340<br>TT = 0.587
BCHE	rs1803274<br>g.68974G>A,<br>p.Ala567Thr	G = 0.890 (0.86-0.91)<br>A = 0.110 (0.09-0.14)	GG = 0.786<br>GA = 0.207<br>AA = 0.007
	rs1355534<br>g.54397A>G	A = 0.312 (0.28-0.35)<br>G = 0.688 (0.65-0.72)	AA = 0.107<br>AG = 0.410<br>GG = 0.483
CHAT	rs2177370<br>g.26734A>G	A = 0.285 (0.25-0.32)<br>G = 0.715 (0.68-0.75)	AA = 0.097<br>AG = 0.377<br>GG = 0.526
	rs3793790<br>g.28596G>A	G = 0.140 (0.11-0.17)<br>A = 0.860 (0.83-0.89)	GG = 0.017<br>GA = 0.247<br>AA = 0.736
CHRNA7	rs6494223<br>g.4389397C>T	C = 0.529 (0.49-0.57)<br>T = 0.471 (0.43-0.51)	CC = 0.301<br>CT = 0.455<br>TT = 0.243
POR	rs1057868<br>g.75587C>T,<br>p.Ala503Val	C = 0.741 (0.70-0.77)<br>T = 0.259 (0.22-0.30)	CC = 0.548<br>CT = 0.387<br>TT = 0.065

Table S2. Allele and genotype frequencies of genetic variants in Mexican Mestizos with Alzheimer's Disease

Gene (n*)	Variant	Allele frequency	Genotype frequency
APOE (66)	rs7412 g.8041C>T<br>rs429358 g.7903T>C	ε3 = 0.894<br>ε4 = 0.106	ε3/ε3 = 0.788<br>ε3/ε4 = 0.212
ABCB1 (62)	rs1128503 g.167964T>C	C = 0.492<br>T = 0.508	CC = 0.242<br>CT = 0.500<br>TT = 0.258
	rs1045642<br>g.208920T>C	C = 0.613<br>T = 0.387	CC = 0.355<br>CT = 0.516<br>TT = 0.129
	rs2032582<br>g.186947T>G/A	A = 0.040<br>T = 0.395<br>G = 0.581	AG = 0.032<br>AT = 0.048<br>TT = 0.177<br>TG = 0.355<br>GG = 0.387
ACHE (67)	rs1799806<br>g.9884C>G, p.Pro592Arg	C = 0.239<br>G = 0.761	CC = 0.060<br>CG = 0.358<br>GG = 0.582
	rs17884589<br>g.4831C>T	C = 0.734<br>T = 0.261	CC = 0.537<br>CT = 0.403<br>TT = 0.060
	rs10953305<br>g.5974C>T	C = 0.194<br>T = 0.806	CC = 0.089<br>CT = 0.209<br>TT = 0.701
BCHE (67)	rs1803274 g.68974G>A,<br>p.Ala567Thr	G = 0.858<br>A = 0.142	GG = 0.746<br>GA = 0.224<br>AA = 0.030
	rs1355534 g.54397A>G	A = 0.231<br>G = 0.768	AA = 0.060<br>AG = 0.343<br>GG = 0.597
CHAT (68)	rs2177370<br>g.26734A>G	A = 0.397<br>G = 0.603	AA = 0.147<br>AG = 0.500<br>GG = 0.353

	rs3793790<br>g.28596G>A	G = 0.213<br>A = 0.787	GG = 0.073<br>GA = 0.279<br>AA = 0.647
CHRNA7 (114)	rs6494223<br>g.4389397C>T	C = 0.667<br>T = 0.333	CC = 0.509<br>CT = 0.417<br>TT = 0.175
POR (60)	rs1057868<br>g.75587C>T,<br>p.Ala503Val	C = 0.725<br>T = 0.275	CC = 0.517<br>CT = 0.387<br>TT = 0.066
CYP3A5 (59)	*CYP3A5*3*<br>rs776746	A = 0.135<br>G = 0.864	AA = 0.000<br>AG = 0.271<br>GG = 0.729
	*CYP3A5*6*<br>rs10264272	G = 0.974<br>A = 0.025	GG = 0.949<br>GA = 0.051<br>AA = 0.000
NR1I2 (92)	rs2461817<br>c.-22-1425A>C	A = 0.609<br>C = 0.391	AA = 0.380<br>AC = 0.456<br>CC = 0.163
	rs7643645<br>c.-22-579A>G	A = 0.446<br>G = 0.554	AA = 0.206<br>AG = 0.478<br>GG = 0.315
	rs2276707<br>c.827-17C>T	C = 0.859<br>T = 0.141	CC = 0.728<br>CT = 0.261<br>TT = 0.011
	rs3814055<br>c.-1135C>T	C = 0.571<br>T = 0.429	CC = 0.315<br>CT = 0.511<br>TT = 0.174
	rs3814058<br>c.*1232T>C	T = 0.859<br>C = 0.141	TT = 0.728<br>TC = 0.261<br>CC = 0.011

*The genotyping of the pharmacogenetic variants was performed according to the pharmacological treatment prescribed in each patient and considering the sample availability.

Table S3. Evaluation of *ABCB1*, *ACHE*, *BCHE*, *CHAT*, and *CHRNA7* genetic variants with MMSE1 score

Gene	Variant	p Value*
APOE	rs7412	0.837
ABCB1	rs1128503	0.717
	rs1045642	0.553
	rs2032582	0.973
ACHE	rs1799806	0.272
	rs17884589	0.801
	rs10953305	0.374
BCHE	rs1803274	0.012
	rs1355534	0.014
CHAT	rs2177370	0.105
	rs3793790	0.636
CHRNA7	rs6494223	0.097

*Kruskal-Wallis rank sum test

Table S4. Evaluation of non-genetic variables in the first evaluated MMSE score

Variable	p Value*
Sex	0.034
Age	0.965
AD type	0.920
Onset age	0.764
Evol years	0.343
Scholarship, years	0.002
Late/early onset	0.172
Depression	0.938
SAH	0.331
T2DM	0.953

*Kruskal-Wallis rank sum test, or Spearman's Rank Correlation Test. AD, Alzheimer´s disease; SAH, systemic arterial hypertension; T2DM, type 2 diabetes mellitus.

Table S5. Association of CYP2D6 predicted phenotype with the response to donepezil, galantamine, and rivastigmine among patients with Alzheimer's disease.

Group	Non-Responders PM/EM/UM	Responders PM/EM/UM	p Value
All n=69	0.267/0.700/0.033	0.167/0.667/0.167	0.210
Donepezil n=44	0.158/0.789/0.053	0.200/0.667/0.133	0.650
Galantamine n=17	0.429/0.571/0.000	0.167/0.667/0.167	0.409
Rivastigmine n=8	0.500/0.500/0.000	0.000/0.667/0.333	1.000
